# Application of nerve transfer for shoulder reanimation in brachial plexus injury

**Published:** 2012-11

**Authors:** Mohammadreza Emamhadi

**Affiliations:** ^a^Poursina Hospital, Gilan University of Medical Sciences, Rasht, Iran.

## Abstract

**Background:**

The present study was aimed to evaluate the functional outcome and practical effectiveness of spinal accessory to suprascapular nerve transfer (XI-SSN) performed for restoration of shoulder reanimation.

**Methods:**

This was a prospective study conducted on 10 consecutive cases of brachial plexus injury during December 2010 to October 2011.The patients aged 16 to 47 years old (mean age 23 years). The injury-surgery interval was between 3-5months (average 4.2 months). All patients underwent XI-SSN nerve transfer. The follow-up period was 5-10 months average 7.2 months (.

**Results:**

Of 10 patients, 8 cases showed good/excellent results in terms of shoulder function. The function evaluation of the shoulder revealed that two cases had M2, three M3, and five cases had M4 shoulder abduction. The average range of shoulder abduction was 52 ° (range 43-65 °).

**Conclusions:**

Spinal accessory to suprascapular nerve transfer (XI-SSN) can be a good treatment option for restoration of shoulder abduction. In our patients, shoulder abduction assessments showed a high rate of recovery such that more than half of the patients can be expected to have active movements. However, to reach a definitive conclusion regarding the treatment efficacy of the XI-SSN technique, longer period follow-ups are necessary.

**Keywords:**

Brachial plexus injury, Nerve transfer, Suprascapular nerve transfer, Shoulder reanimation, Spinal accessory


					Volume 4, Suppl. 1 Nov 2012
Publisher: Kermanshah University of Medical Sciences
URL: http://www.jivresearch.org
Copyright:
Congress of Iranian Neurosurgeons, 14-16 November 2012, Kermanshah, Iran
Paper No. 9
Application of nerve transfer for shoulder reanimation in
brachial plexus injury
Mohammadreza Emamhadi a,*
a
Poursina Hospital, Gilan University of Medical Sciences, Rasht, Iran.
Abstract:
Background: The present study was aimed to evaluate the functional outcome and practical effectiveness of spinal
accessory to suprascapular nerve transfer (XI-SSN) performed for restoration of shoulder reanimation.
Methods: This was a prospective study conducted on 10 consecutive cases of brachial plexus injury during December
2010 to October 2011.The patients aged 16 to 47 years old (mean age 23 years). The injury-surgery interval was
between 3-5months (average 4.2 months). All patients underwent XI-SSN nerve transfer. The follow-up period was
5-10 months average 7.2 months( .
Results: Of 10 patients, 8 cases showed good/excellent results in terms of shoulder function. The function evaluation
of the shoulder revealed that two cases had M2, three M3, and five cases had M4 shoulder abduction. The average
range of shoulder abduction was 52  (range 43-65 ).
Conclusions: Spinal accessory to suprascapular nerve transfer (XI-SSN) can be a good treatment option for
restoration of shoulder abduction. In our patients, shoulder abduction assessments showed a high rate of recovery
such that more than half of the patients can be expected to have active movements. However, to reach a definitive
conclusion regarding the treatment efficacy of the XI-SSN technique, longer period follow-ups are necessary.
Keywords:
Brachial plexus injury, Nerve transfer, Suprascapular nerve transfer, Shoulder reanimation,
Spinal accessory
*Corresponding Author at:
Mohammadreza Emamhadi: Associate Professor of Neurosurgery, Poursina Hospital, Gilan University of Medical Sciences, Rasht, Iran.
Phone: +98(911)-2482003, Email: imamhady@yahoo.com, (Emamhadi M.).

				

